# Supplementary material for: Calomys callosus: An Experimental Animal Model Applied to Parasitic Diseases Investigations of Public Health Concern
Source: Pathogens. 2022 Mar 17;11(3):369. doi: 10.3390/pathogens11030369 (PMC8948650; doi:10.3390/pathogens11030369)
Supplement: Supplementary file 1 [file pathogens-11-00369-s001.zip › pathogens-1603289-supplementary.pdf]

Table S1. Current findings using *C. callosus* as an experimental model to study parasitic diseases.

Parasite	Strain	Animal age	Dose	Route of infection	Infection time	Major findings	Reference
Toxoplasma gondii	RH	8 weeks old	10^2, 10^3, 10^4, 10^5 and 10^6 tachyzoites	Intraperitoneal	3 and 9 days	*C. callosus* demonstrated to be highly susceptible to parasitic infection; massive presence of extracellular parasites in the peritoneal cavity, and high parasite load in spleen, lung, intestine, brain, and kidney	23
	RH	12 weeks old	10^5 tachyzoites	Intraperitoneal	1, 3, 6, 12, 24, 36 and 48 hours	Mast cells are an important cell type involved with the acute phase of the inflammatory response against *T. gondii*	24
	TgChBrUD1 and TgChBrUD2	-	10^2 tachyzoites	Intraperitoneal	8 and 15 days	*C. callosus* is susceptible to parasite infection with gender differences in severity of infection, regardless of *T. gondii* strain	25
	ME49	8-12 weeks old	20 cysts	Oral	15, 16, 17, 18, 19 and 21 days	Males and virgin females (acquired form), and pregnant females (congenital form) of *C. callosus* developed severe ocular lesions with presence of cysts, free tachyzoites and inflammatory cells in the retina	32
	RH	60 days old	10^2 or 10^3 tachyzoites	Intraperitoneal or conjunctival	24 hours and 5 days	Enucleated eyes of *C. callosus* presented tachyzoites, inflammatory cells and vasodilatation after parasite infection	33
	RH	12-16 weeks old	10^2 tachyzoites	Intraperitoneal	7 days after pregnancy	*T. gondii* is capable of infecting trophoblast cells in the early stage of pregnancy of *C. callosus*	38
	ME49	12-16 weeks old	20 cysts	Oral	100 days and 20 days after pregnancy	Vertical transmission of *T. gondii* occurs in acutely infected pregnant of *C. callosus*, but not in the chronic phase of the infection	11
	ME49	-	20 cysts	Oral	17 and 20 days after pregnancy	Susceptibility to vertical transmission of *T. gondii* is temporally dependent on the preconceptional infection in *C. callosus*	39
	ME49, RH, TgChBrUD1 and TgChBrUD2	-	20 cysts; 10^2 tachyzoites	Oral or intraperitoneal	19 days after pregnancy	*C. callosus* females chronically infected with *T. gondii* when reinfected during pregnancy resulted in the vertical transmission	46
	ME49	-	20 cysts	Oral	35 days	The antibiotic enrofloxacin diminished the tissue parasitism as well as the inflammatory alterations in the brain of *C. callosus* infected with *T. gondii*	57
	ME49	12 weeks old	20 cysts	Oral	15 and 20 days after pregnancy	Azithromycin inhibited	55

						the vertical transmission of *T. gondii* in females of *C. callosus*	
	ME49	12-16 weeks old	20 cysts	Oral	15 and 19 days after pregnancy	Azithromycin reduced the parasite load in the brain of mothers, and no parasites were detected in eyes of fetuses from *C. callosus*	56
Leishmania amazonensis	Isolate human case (Brasília, Brasil)	2-3 months old	0.1 ml of tritu of amastigote lesions	Subcutânea	3,5 month	The lesions manifested at the application sites from the 40th day post-infection (dpi) and the presence of the parasite was confirmed by smears of these tissues	16
Leishmania infantum	Isolate human case (Maranhão, Brasil)	2 months old	0.1 ml of a homogenate in sterile saline, from a homologous animal's spleen, rich in amastigote shapes	*	3 month	Animals low body weight. Splenomegaly was also recorded. "*In vitro*" cultures were positive liver and spleen in 67% of the animals. Blood tests were negative. Histological studies of unaltered liver Kupffer cell proliferation and granulomatous reaction in portal areas with multinucleated cells and amastigote forms of the parasites. Loss of follicular pattern with parasitism in a large number of cells around which where granulomatous reactions were observed in the spleen	17
Trypanosoma cruzi	R52, R64, R65 e M226	34-48 days old	0,1 ml of solution (ratio: 50% barber bug fecal material and 50% saline)	Intraperitoneally	63 days	The results obtained in the experiments performed with *C. callosus* are important data in demonstrating the ability of the species to maintain a patent, regular and long parasitemia for the four strains studied. *C. callosus* inoculated with the three strains (R52, R64 and R65) were more sensitive in acquiring the infection, with parasitemia peaks of up to 7,632 parasites/5mm³, even with low lethality, while for the M226 strain the parasitemia peaks were low and lethality zero.	74
	R52, R64, R65 e M226	26-36 days old	10^4 metacyclic trypomastigotes	Intraperitoneally	6 and 18 days	Although it is believed that the maintenance of *T. cruzi* "in vitro" contributed to the loss of infective power and virulence of the pathogen, the results obtained showed that there was no complete loss of infective capacity, since *C. callosus* showed positive results, high parasitemia although irregular in some animals, and prepatency	75

						between 6.0 to 8.6 days. The lethality in these animals was zero.	
	R52, R64 e R65	*	10.000 trypomastigote	Intraperitoneally	30-60 days	Female *C. callosus* were infected with *T. cruzi* trypomastigote. Upon reaching the chronic phase, these females started to breed and 90 neonates were analyzed by blood tests and xenodiagnosis. The results obtained were positive for infection even with a low parasitemia in the neonates, however, it was not possible by the nature of the experiments to determine whether *T. cruzi* was transmitted to the neonates via transplacental or breast milk.	83
Trypanosoma lewis	*	*	0.1 ml. of blood infected	Intraperitoneally	45-60 days	All experimentally inoculated *C. callosus* were infected, presenting a high parasitemia while the attempts to infect other rodents were unsuccessful. The results of experimental inoculations suggest a high degree of host specificity of *T.lewis* and validate the trypanosome described.	79
	Isolate human case (Pernambuco, Brazil)	2-3 months old	45 cercarie	Transcutaneous	50 days	The animals were positive in the coprological exams, up to the 50th day after inoculations. The coprological evaluation also applies that the pre-patent period took place between 43 to 44 days	16
	B. glabrata-BH	5 days old	70 cercarie	Percutaneous	42,45,55.80.90 and 160 days	Infected animals presented fibrovascular lesions found in the intestinal subserosa region between 55 and 160 days after infection.	93
	B. glabrata-BH	5 days old	70 cercarie	Percutaneous	42 days	Infected *C. callosus* developed prominent intestinal subserous nodules with diffuse granular features	94
Schistosoma mansoni	*	5 days old	70 cercarie	Percutaneous	42,55, 90, 160 and 229	Omental milky spots from infected animals were found in lymphomyelocytic cells between 42 and 90 days after infection and lymphoplasmocytic cells 160 days after infection. Lymphoid follicles with germinal centers, plasmacytogenesis and plasmacytosis, mastocytosis, megakaryopoiesis, erythropoiesis and less eosinopoiesis were also observed.	97
